# Prevalence of traditional cardiovascular risk factors among Nigerians with stroke

**Published:** 2007-07

**Authors:** KM Karaye, I Nashabaru, GM Fika, DA Ibrahim, BM Maiyaki, NA Ishaq, LY Abubakar, AM Nalado, M Hassan, AK Bello, SM Yusuf

**Affiliations:** Department of Medicine, Bayero University/Aminu Kano Teaching Hospital, Kano, Nigeria; Department of Medicine, Bayero University/Aminu Kano Teaching Hospital, Kano, Nigeria; Department of Medicine, Bayero University/Aminu Kano Teaching Hospital, Kano, Nigeria; Department of Medicine, Bayero University/Aminu Kano Teaching Hospital, Kano, Nigeria; Department of Medicine, Bayero University/Aminu Kano Teaching Hospital, Kano, Nigeria; Department of Medicine, Bayero University/Aminu Kano Teaching Hospital, Kano, Nigeria; Department of Medicine, Bayero University/Aminu Kano Teaching Hospital, Kano, Nigeria; Department of Medicine, Bayero University/Aminu Kano Teaching Hospital, Kano, Nigeria; Department of Medicine, Bayero University/Aminu Kano Teaching Hospital, Kano, Nigeria; Department of Medicine, Bayero University/Aminu Kano Teaching Hospital, Kano, Nigeria; Department of Medicine, Bayero University/Aminu Kano Teaching Hospital, Kano, Nigeria

## Abstract

Stroke is an important cause of morbidity and mortality worldwide. The case fatality rates from stroke are two- to three-fold higher in sub-Saharan Africa, including Nigeria, than in the developed world, mainly because of limited healthcare facilities and untreated risk factors. The aim was to determine the prevalence of traditional cardiovascular risk factors among Nigerians with stroke and compare the prevalence of risk factors between young and older adults with stroke.

**Methods:**

The study was cross-sectional in design, and was carried out on stroke patients who were 15 years of age or older, in the medical wards and neurology clinic of Aminu Kano Teaching Hospital, Nigeria. Data was collated consecutively over six months.

**Results:**

A total of 81 patients were studied. Sixteen of them (19.8%) were under 45 years old (group 1) while the remaining 65 patients (80.2%) were 45 years or older (group 2). All patients had at least one risk factor. One-third of group 1 patients (37.5%) and 81.5% of group 2 patients had three or more cardiovascular risk factors (*p* = 0.0004). The most widespread risk factor in all patients, particularly in group 2 patients was systemic hypertension, while dyslipidaemia was most common among group 1 patients. Recurrent stroke was significantly more common among group 2 than group 1 patients (30.8 and 6.3% respectively) (*p* = 0.045).

**Conclusion:**

Cardiovascular risk factors, particularly hypertension and dyslipidaemia were prevalent in the studied patients with stroke. The older patients in group 2 had more multiple-risk factors than the younger ones in group 1. Secondary prevention strategies including detection and treatment of risk factors may curtail the burden of the disease.

## Summary

Globally, stroke is the second leading cause of death.^1^ Preliminary studies in sub-Saharan Africa and the Caribbean indicate that case fatality rates from stroke are two- to three-fold higher than those in developed countries, and this has been attributed to limited healthcare facilities and untreated risk factors.^2^ In addition to being a major cause of death, many surviving stroke patients are disabled and need help in activities of daily living, which must be provided by family members, the healthcare system or other social institutions.^1^ This puts further financial strain on the already meagre resources available to both the individuals and states in the West African sub-region.

Patients who have suffered a stroke are at a 30 to 43% increased risk of a further stroke within five years. They also have an increased risk of acute myocardial infarction and other vascular events.^3^ A key component of the secondary prevention strategies for stroke advocated by the World Health Organisation (WHO) is intensified reduction in exposure to major cardiovascular risk factors. These risk factors may be classified into traditional (established) and new (emerging) risk factors. Examples of the former include high blood pressure, dyslipidaemia, diabetes mellitus and cigarette smoking, and account for the majority of stroke events.^4^ Emerging risk factors have been found to be more relevant in the aetiology of stroke in young individuals, and examples include prothrombotic states, infections, vasculitis and emotional stress.^5^

This study therefore aimed at determining the prevalence of traditional cardiovascular risk factors among Nigerians with stroke, as well as comparing the prevalence of the risk factors between young (< 45 years of age) and older (≥ 45 years of age) adults with stroke. The information will then form the basis of our recommendations for alleviating the burden of the disease.

## Methods

The study was carried out in Aminu Kano Teaching Hospital (AKTH), which is affiliated to Bayero University Kano, and is the only tertiary medical centre in the most populous Nigerian state of Kano.

The study was cross-sectional in design. The AKTH research ethics committee approved the protocol of the study before its commencement. Patients were enrolled consecutively into the study by the authors over a six-month period beginning in October 2006. All patients with the diagnosis of stroke who were hospitalised in the medical wards of the hospital within this period, or their first-degree relations, gave informed consent for participating in the study and were recruited. Similarly, all patients with stroke who attended the neurology clinic of the hospital within the study period, or their first-degree relations, also gave informed consent for participating in the study and were recruited. Patients were enrolled into the study only if they were 15 years of age or older. The principal author (KMK) was responsible for collation of all the data and ensured its quality.

Stroke was defined according to the standard WHO recommendation as ‘a focal (or at times global) neurological impairment of sudden onset, and lasting more than 24 hours (or leading to death), and of presumed vascular origin’.^1^ This definition is based on clinical information and has been validated to be reliable. 6 The diagnosis of stroke was confirmed with brain computed tomographic (CT) scans whenever feasible. Recurrent stroke was also defined according to standard WHO criteria.^1^ The assessed cardiovascular risk factors for stroke were recognised by the WHO^1^ and American Heart Association/American Stroke Association (AHA/ASA).^7^ The assessed risk factors were: male gender; elevated blood pressure (BP); diabetes mellitus (DM); current tobacco smoking; heavy alcohol consumption; recurrent stroke; heart diseases such as atrial fibrillation (AF), valvular heart disease and cardiomyopathies; dyslipidaemia; and use of oral contraceptive pills (OCP) in women.

A history was taken from patients or their first-degree relations. All patients were also examined physically, and all had ECG and other available baseline investigations. Further evaluation included a brain CT scan and when indicated, trans-thoracic echocardiography. Other investigations were only carried out if indicated.

Elevated BP was defined according to the recommendations of the WHO/International Society of Hypertension (ISH), using systolic BP (SBP)/diastolic BP (DBP) cut-off values of ≥ 140/90 mmHg.^8^ Diabetes mellitus was defined according to the WHO criteria.^9^ Heavy alcohol consumption was defined as the intake of more than five alcoholic drinks per day.^7^ Dyslipidaemia was defined as the presence of any of high total cholesterol (TC) (> 5.2 mmol/l), high low-density lipoprotein cholesterol (LDL-C) (> 3.38 mmol/l) or low high-density lipoprotein cholesterol (HDL-C) (< 1.0 mmol/l). These cut-off values were based on the Adult Treatment Panel III (ATP III) recommendations for borderline-high cholesterol (TC and LDL-C) and low HDL-C respectively.^10^ The current use of OCP for at least six months before the stroke was counted as a risk factor.

Data analysis was done using SPSS version 10.0. Means and standard deviations were computed for quantitative variables. The Chi-squared or Fisher’s exact tests were used to test for significance of observed associations, and the Student’s *t*-test was used to compare means. A *p*-value of < 0.05 was considered significant.

## Results

A total of 81 patients with stroke were studied, comprising both in- and out-patients. Forty-two of them (51.9%) were males and thirty-nine (48.1%) were females; the male:female ratio was 1.1:1.0. The mean age of all patients was 55.83 ± 16.15 years, with an age range of 17 to 85 years.

Subjects were placed into two groups: patients under 45 years old were in group 1 and those aged 45 years and older were in group 2. There were 16 patients (19.8%) in group 1 and 65 (80.2%) in group 2. Overall, stroke was confirmed with brain CT scan in only six patients (7.4%) who could afford the procedure. Three of these patients had cerebral infarction, two others had intracerebral haemorrhage and the last one had subarachnoid haemorrhage. The clinical diagnosis of stroke and subarachnoid haemorrhage agreed with the CT scan findings. Other baseline data on the two groups of patients are presented in Table 1.

**Table 1.** Baseline Characteristics Of Patients

*Characteristic*	*Group 1 n = 16*	*Group 2 n = 65*	p*-value*
Mean age (years)	31.19 ± 8.20	61.89 ± 11.00	< 0.0001*
Mean SBP (mmHg)	142.19 ± 43.93	155.23 ± 28.29	0.146
Mean DBP (mmHg)	92.50 ± 26.46	94.81 ± 22.13	0.719
Mean TC (mmol/l)	5.54 ± 1.21	5.20 ± 1.18	0.307
Mean HDL-C (mmol/l)	1.01 ± 0.02	1.09 ± 0.27	0.224
Mean LDL-C (mmol/l)	3.89 ± 1.08	3.51 ± 0.89	0.160
Mean serum Cr (μmol/l)	103.56 ± 110.57	122.74 ± 152.41	0.642
Mean FPG (mmol/l)	4.59 ± 0.53	6.07 ± 2.26	0.096

*n*, number of patients; SBP, systolic blood pressure; DBP, diastolic blood pressure; TC, total cholesterol; HDL-C, high-density lipoprotein cholesterol; LDL-C, low-density lipoprotein cholesterol; Cr, creatinine; FPG, fasting plasma glucose. *p-value statistically significant. Results are given as values ± standard deviation.

The prevalence of the various cardiovascular risk factors in the two groups of patients is presented in Table 2. It shows that all patients had at least one risk factor. Whereas the majority of patients in group 2 (81.5%) had three or more risk factors, only one-third of group 1 patients had such numbers of risk factors (37.5%) (*p* = 0.0004). It also shows that the commonest risk factor was history of systemic hypertension, found in 79% of all patients and 89.2% of group 2 patients. However, only about a third of group 1 patients (37.5%) had a history of hypertension.

**Table 2.** Vascular Risk Factors In Groups 1 And 2 Patients With Stroke

*Risk factors*	*Group 1 n = 16 (%)*	*Group 2 n = 65 (%)*	p*-value*	*Total n = 81 (%)*
Male gender	8 (50)	34 (52.3)	0.869	42 (51.9)
Total of RF:			0.0004*	
1−2	10 (62.5)	12 (18.5)		22 (27.2)
≥ 3	6 (37.5)	53 (81.5)		59 (72.8)
History of hypertension	6 (37.5)	58 (89.2)	< 0.0001*	64 (79.0)
History of DM	0	9 (13.9)	−	9 (11.1)
Tobacco smoking	0	7 (10.8)	−	7 (8.6)
Excess alcohol	0	1 (1.5)	−	1 (1.2)
Dyslipidaemia	13 (81.3)	44 (67.7)	0.287	57 (70.4)
Heart diseases	5 (31.3)	48 (73.9)	0.0013*	53 (65.4)
Atrial fibrillation	0	1 (1.5)	–	1 (1.2)
Use of OCP	2 (12.5)	0	–	2 (2.5%)
Recurrent stroke	1 (6.3)	20 (30.8)	0.045*	21 (25.9)

*n*, number of patients; RF, risk factors; DM, diabetes mellitus; OCP, oral contraceptive pills. *p-values statistically significant.

The second commonest risk factor in all patients (65.4%) as well as in group 2 patients (73.9%) was heart disease. Again, only about a third of group 1 patients (31.3%) were found to have heart disease; all had hypertensive heart disease. Hypertensive heart disease was also the most common heart disease found among group 2 patients, affecting 44 of them (67.7%). Dilated and non-dilated cardiomyopathies as well as aortic valve sclerosis were isolated findings among group 2 patients. Two patients in group 2 were found to have premature atrial extra-systoles, while three other patients in the same group had ventricular extra-systoles. Atrial fibrillation was found in only one patient in group 2.

Trans-thoracic echocardiography was requested and performed on a total of 32 patients (39.5%), and intracardiac thrombus was not found in any of them. The most frequent vascular risk factor among group 1 patients was dyslipidaemia, affecting 81.3% of them, while it was found among two-thirds of group 2 patients (67.7%) (*p* = 0.287).

The frequency of recurrent stroke among group 2 patients was high, affecting up to one-third of them (30.8%). It was however rare in group 1 patients and found in one patient only (6.3%) (*p* = 0.045). Although only about one-third of patients in group 1 had a history of systemic hypertension, their mean SBP and DBP were high and not significantly different from mean BPs of patients in group 2 (*p* = 0.146 and 0.719 for SBP and DBP, respectively).

The means of fasting plasma glucose (FPG) for patients in the two groups are presented in Table 1. The FPG of patients in group 2, after excluding patients with DM, was 5.24 ± 0.91 mmol/l and a comparison with mean FPG for group 1 patients revealed no significant statistical difference (*p* = 0.080). In addition, chronic renal failure was found in a total of four patients (4.94%); one of them in group 1 (6.3%) and the remaining three in group 2 (4.6%) (*p* = 0.787).

## Discussion

In this study, we have reported on the prevalence of traditional cardiovascular risk factors among Nigerians with stroke, and compared the prevalence between young and older adults.

In developed countries, stroke is a disease of the elderly.^11^ The mean age of patients in our study was 56 years. Similarly, patients with stroke in Maiduguri (north-eastern Nigeria)^12^ had a mean age of 55 years, whereas stroke patients in Ogun and Lagos States (south-western Nigeria)^13^ had a mean age of 54 years. It would therefore appear that in a developing country like Nigeria, patients with stroke are younger than their counterparts in developed countries, perhaps a reflection of the life expectancy of its citizens. The WHO recently reported that the life expectancy at birth for Nigerians was 47 and 48 years for males and females, and 75 and 80 years for males and females in the USA, respectively.^14^ However, the majority of our patients (80.2%) actually belonged to group 2 (patients aged 45 years or older), with a mean age of 61.9 years. Therefore stroke was more common among the older patients in our series.

In the present study, there were equal numbers of males and females affected by stroke in group 1, whereas in group 2 stroke was slightly more common in males. Stroke was similarly reported to be more common among males in other regions in Nigeria^13^ and other parts of the world such as the USA,^11^ Burkina Faso^15^ and the United Kingdom.^16^

In the present study, all the patients had at least one risk factor and the majority of them (73%) had at least three risk factors. In group 1 however, only one-third of patients had three or more risk factors. Other than male gender, the most frequent risk factors among group 1 patients were modifiable, namely dyslipidaemia, hypertension and hypertensive heart disease.

In a retrospective study on stroke patients in Saudi Arabia, young patients (aged 15−45 years) with stroke constituted 12.7% of all patients, and hypertension was the main risk factor for intracerebral haemorrhage (26%).^17^ However, some unusual risk factors and causes of stroke in the young were also found, including thoracic outlet syndrome with retrograde embolism, vascular malformations and dissecting arterial aneurysms. Other studies in Italy,^18^ Burlington (USA)^19^ and Nancy (France)^20^ all revealed the significance of uncommon risk factors and diseases in the aetiology of stroke in the young.

Our study aimed at assessing the pattern of some of the established cardiovascular risk factors (mentioned above) among stroke patients, but we acknowledge the fact that some uncommon risk factors are important in the aetiology of stroke, especially in the younger population.

In this study, two patients (25% of the females) in group 1 were on OCP. This result is higher than what was reported from an Italian study^18^ (15.3% of young women), but less than what was reported from Nancy^20^ (53% of young women) and Switzerland^21^ (66% of young women).

Elevated BP is a powerful determinant of stroke risk. Individuals with BP less than 120/80 mmHg have about half the lifetime risk of stroke of subjects with hypertension.^22^ The importance of systemic hypertension in the aetiology of stroke has been well described among Nigerians and other black West Africans, from the works of Osuntokun^23^ to those of several others.^12^,^15^ In our study, systemic hypertension was the most frequent risk factor among all patients (79%) as well as among group 2 patients (89%). These results are similar to reports from Maiduguri (79%)^12^ and Burkina Faso (84%).^15^

Although a history of hypertension was significantly more frequent among patients in group 2 than in group 1 (*p* < 0.0001), the means of SBP and DBP of patients in both groups of our study were high and similar (*p* = 0.146 and 0.719 for SBP and DBP, respectively). This suggested a higher frequency of undiagnosed pre-existing systemic hypertension among group 1 patients. A previous study reported that 64% of stroke patients with hypertension were unaware of their hypertension.^12^ Furthermore, elevated BP among patients with acute stroke has been correlated with a past history of hypertension.^24^

Hypercholesterolaemia or dyslipidaemia are not as well established risk factors for first or recurrent stroke as they are in ischaemic heart disease, but the assessment of serum lipids is recommended in all stroke patients.^7^ In our study, dyslipidaemia was the second most frequent risk factor among all patients combined (70%). It was also the most frequent risk factor among group 1 patients (in 81%) and the third most frequent among group 2 patients (in 68%). Two reports from the USA similarly revealed high frequencies of dyslipidaemia of 50.7%^25^ and 58.8%^26^ among stroke patients, but the frequency reported from Burkina Faso was lower (20.6%).^15^ The frequency of dyslipidaemia among our young patients with stroke was higher than what was reported from Nancy (35%) among young patients with stroke.^20^ Our definition of dyslipidaemia might have contributed to the high frequency we obtained. However, a previous national survey in Nigeria^27^ revealed that the residents of Kano had the highest serum cholesterol levels (and systemic hypertension) in the country.

In our series, about one-quarter of all patients and one-third of group 2 patients had recurrent stroke. This recurrent stroke rate is similar to the report by Bogar *et al.* from the USA (25%),^26^ and also by Truelsen *et al.* (19%)^28^ in a study carried out in nine developing countries across Asia and Africa (including Nigeria). The rate of recurrent stroke in our study is not surprising given the fact that up to 72.8% of our patients had at least three vascular risk factors. The majority of our patients were hypertensive, and hypertensive patients in Kano were previously found to be at very high absolute risk of cardiovascular disease, both during pharmacotherapy (75.6%) and at the time of diagnosis (68.5%).^29^

Heart diseases (principally hypertensive heart disease) were found among about two-thirds of all patients. The frequencies of a history of hypertension and of heart disease were similar in both groups of patients. In the first instance, this demonstrates the role of hypertension in the development of heart disease. It also suggests that in individual patients, the hypertension had been present for such a long time as to have caused end-organ damage in the heart. Hypertensive heart disease is a risk factor for stroke; it predisposes to the development of left ventricular hypertrophy, cardiac arrhythmia, heart failure, myocardial ischaemia, left atrial abnormalities and functional valvular regurgitation.^30^,^31^

Histories of tobacco smoking and DM were absent among group 1 patients and uncommon among group 2 patients in our series (10.8 and 13.9%, respectively). A similar pattern was reported from north-eastern Nigeria (frequency of DM of 8%)^12^ and Burkina Faso (frequency of DM of 7.3%, smoking 12.4%) among patients with stroke,^15^ but a higher rate of smoking (61%) among stroke patients was reported from Nepal (India)^32^ although that of DM was 8%. The frequency of DM among stroke patients was higher in the USA, in the order of 15 to 33%.^7^ The frequencies of tobacco smoking and DM among all patients in our study were 8.6 and 11.1%, respectively. This frequency of smoking was similar to the prevalence of smoking in the general Nigerian population (8.9%), but that of DM was significantly higher than the prevalence rate of DM (2.2%) in the general Nigerian population.^27^

Atrial fibrillation and a history of excessive alcohol intake were isolated findings in the present study. Alcohol intake is not a culturally acceptable habit among the people of Kano, hence its rarity in our series. Atrial fibrillation (AF) was found in one patient in group 2 (1.5%). This frequency of AF in our series was similar to the reported frequency of AF among patients with stroke from Canada (2%)^33^ and the United Kingdom (0.08%),^34^ but lower than what was reported from India (8%).^32^

An important limitation in our study was the small number of patients in group 1. A longer study that may yield a larger sample size would be desirable. However, the frequent industrial strike actions by labour and professional unions in Nigeria make long-term studies difficult to carry out. Another limitation is the cost of brain CT scans at the study centre (about US $190−195), which is affordable to only a few individuals. However, we felt that we should not allow these limitations to preclude us from carrying out scientific research, although regrettably they do limit the impact of the study.

## Conclusion

Nigerians with stroke attending AKTH were found to have various vascular risk factors for stroke and the majority had at least three such risk factors. The older patients had a significantly higher frequency of multiple risk factors. The most frequent risk factor among all patients combined, as well as among the older patients was hypertension. The most frequent risk factor among the younger patients was dyslipidaemia, followed by hypertension. The rate of recurrent stroke among group 2 patients was significant.

We recommend that special attention be paid to the secondary prevention of stroke, particularly to the treatment of hypertension and dyslipidaemia. We also recommend that the cost of drugs for the treatment of hypertension and dyslipidaemia, and that for such investigations as brain CT scans be subsidised by the governments in conjunction with the allied industries. Furthermore, the provision and maintenance of good-quality equipment will improve the overall care of patients.
